# P-1232. PK/PD Analysis and Dose Optimization of Tebipenem and Faropenem against ESBL-Producing *Enterobacterales* in Patients with UTI Considering Renal Function

**DOI:** 10.1093/ofid/ofae631.1414

**Published:** 2025-01-29

**Authors:** Yukihiro Hamada, Takumi Maruyama, Hidefumi Kasai, Yusuke Yagi, Nobuaki Matsunaga

**Affiliations:** Department of Pharmacy, Kochi Medical School Hospital, NANKOKU, Kochi, Japan; Pharmacist, Shinjuku, Tokyo, Japan; Keio, Kawasaki, Kanagawa, Japan; Kochi medical school hospital, NANKOKU, Kochi, Japan; National Center for Global Health and Medicine, Shinjuku, Tokyo, Japan

## Abstract

**Background:**

Tebipenem (TBPM) and faropenem (FRPM) have been proposed as effective treatments for urinary tract infections caused by ESBL-producing *Enterobacterales*. However, appropriate dosage according to renal function needs to be considered. This study utilized PK/PD analysis to investigate the optimal dosage.

**Methods:**

Monte Carlo simulation using the model of Nakashima et al, 2009 for TBPM and Hirooka et al. 2005, for FRPM generated blood concentration profiles for 1000 cases stratified by renal function. The optimal dose metric was defined as the Probability of Target Attainment (PTA) of 90 percent or more for PK/PD parameters at the MIC90. The PK/PD parameters for TBPM and FRPM were defined as 24h free AUC/MIC/dosing interval 34.58 or more, and free time above MIC 20 and 40 percent or more. The MIC90 values were set at 0.03 and 2 mg/L, respectively.

**Results:**

For TBPM, the dose achieving PTA of 90 percent or more was 150 mg (q12h) for creatinine clearance (Ccr) less than 30 and 300 mg (q8h) for Ccr 30 or more and less than 80 mL/min. For Ccr 80 mL/min or more was not achieved. None of the doses for FRPM achieved PTA of 90 percent or more.

**Conclusion:**

The dose for TBPM suggested the need for an even higher dose for Ccr 80 mL/min or more. Cases of post-treatment relapse have been reported for FRPM. Therefore, the optimal dosage considering PK/PD analysis with urinary concentrations needs further validation.

**Disclosures:**

**All Authors**: No reported disclosures

